# Multi-criteria decision analysis and spatial statistic: an approach to determining human vulnerability to vector transmission of *Trypanosoma cruzi*

**DOI:** 10.1590/0074-02760160523

**Published:** 2017-10

**Authors:** Diego Montenegro, Ana Paula da Cunha, Simone Ladeia-Andrade, Mauricio Vera, Marcel Pedroso, Angela Junqueira

**Affiliations:** 1 Fundação Oswaldo Cruz-Fiocruz, Instituto Oswaldo Cruz, Laboratório de Doenças Parasitárias, Rio de Janeiro, RJ, Brasil; 2 Fundación Chilloa, Santa Marta, Colombia; 3 Fundação Oswaldo Cruz-Fiocruz, Instituto de Comunicação e Informação Científica e Tecnologia em Saúde, Rio de Janeiro, RJ, Brasil; 4 Ministerio de Salud y de la Protección Social, Bogotá, Colombia

**Keywords:** vulnerability, Chagas disease, multi-criteria decision analysis, spatial statistic, PROMETHEE method, GAIA method

## Abstract

**BACKGROUND:**

Chagas disease (CD), caused by the protozoan *Trypanosoma cruzi*, is a neglected human disease. It is endemic to the Americas and is estimated to have an economic impact, including lost productivity and disability, of 7 billion dollars per year on average.

**OBJECTIVES:**

To assess vulnerability to vector-borne transmission of *T. cruzi* in domiciliary environments within an area undergoing domiciliary vector interruption of *T. cruzi* in Colombia.

**METHODS:**

Multi-criteria decision analysis [preference ranking method for enrichment evaluation (PROMETHEE) and geometrical analysis for interactive assistance (GAIA) methods] and spatial statistics were performed on data from a socio-environmental questionnaire and an entomological survey. In the construction of multi-criteria descriptors, decision-making processes and indicators of five determinants of the CD vector pathway were summarily defined, including: (1) house indicator (HI); (2) triatominae indicator (TI); (3) host/reservoir indicator (Ho/RoI); (4) ecotope indicator (EI); and (5) socio-cultural indicator (S-CI).

**FINDINGS:**

Determination of vulnerability to CD is mostly influenced by TI, with 44.96% of the total weight in the model, while the lowest contribution was from S-CI, with 7.15%. The five indicators comprise 17 indices, and include 78 of the original 104 priority criteria and variables. The PROMETHEE and GAIA methods proved very efficient for prioritisation and quantitative categorisation of socio-environmental determinants and for better determining which criteria should be considered for interrupting the man-*T. cruzi*-vector relationship in endemic areas of the Americas. Through the analysis of spatial autocorrelation it is clear that there is a spatial dependence in establishing categories of vulnerability, therefore, the effect of neighbors’ setting (border areas) on local values should be incorporated into disease management for establishing programs of surveillance and control of CD via vector.

**CONCLUSIONS:**

The study model proposed here is flexible and can be adapted to various eco-epidemiological profiles and is suitable for focusing anti-*T. cruzi* serological surveillance programs in vulnerable human populations.

Chagas disease (CD), produced by the protozoan *Trypanosoma (Schizotrypanum) cruzi* (Chagas 1909), is a neglected human disease. It is endemic to the Americas, but cases have been reported from almost every continent of the world (WHO 2015). Approximately 7 million people are infected in Latin America, a region where it causes more than 7,000 deaths per year (WHO 2015). It has been estimated that the economic impact of CD, including lost productivity and disability, averages 7 billion dollars per year (Lee et al. 2013).

Since the discovery of vector transmission of CD by Carlos Chagas (Chagas 1909), which is known as the classical pathway of Chagas pathology, it continues to be considered a zoonosis with multifactorial determinants: different species of triatomine vectors and mammalian reservoirs, infrastructural conditions of housing favorable to the domiciliation of vectors, lack of specific information by those living in areas at risk, low access to health services, lack of vaccines, drugs effective only in acute infections, etc. (Chagas 1909, Forattini 1980, Montenegro et al. 2016). The problem of the disease extends beyond the health sector, and requires holistic policies that integrate the search for cost-effective measures; the performance of sustainable surveillance; and the promotion, prevention and control of the disease, including comprehensive care for individual cases (i.e., diagnosis, treatment and rehabilitation).

Several proposals to address different biological and socio-environmental determinants for human infection with *T. cruzi* by triatomine vectors are available (Vinhaes et al. 2014, Tah et al. 2015, WHO 2015).

The presence, density and degree of synanthropy exhibited by different populations of triatomines in artificial environments and anthropic areas determine the level of danger for the occurrence of cases of vector transmitted CD (Silveira et al. 1984). However, this is not all that is required for the transmission of the parasite from insects to humans, since certain conditions of the landscape are needed at the local level (indoors and peridomiciliar areas), and the knowledge and practices of people exposed to danger can influence their ability to confront the threat of transmission (Tah et al. 2015). Therefore, to better understand the human-parasite-vector relationship and the degree of social vulnerability to infection, disease or death by *T. cruzi*, all elements must be integrated for a better understanding of the relevant biological, ecological and social determinants.

Over the years, various models have been developed for identifying and determining vulnerability and risk of transmission of the vector of *T. cruzi*, integrating biological (Rabinovich et al. 1979, Silveira et al. 1984, Catalá et al. 1997) and biological with socio-environmental determinants (Guhl 2000, Silveira 2003). However, there is little evidence of the actual implementation of these models, for exemplo in Guhl et al. (2005).

A detailed analysis found that the risk models currently in use (Guhl 2000, Silveira 2003) cannot be applied to areas below the scale of municipalities, such as rural areas and villages. They prioritize the inclusion of an anti-*T. cruzi* serological indicator for human populations in their mathematical algorithms, making them very expensive and not easily applied in routine monitoring programs of CD endemic countries.

Therefore, given the current international effort to interrupt domiciled vectors of *T. cruzi* (WHO 2015) and to strengthen programs of surveillance and vector control in endemic countries, new strategies and methodologies have become necessary so as to integrate the greatest possible number of determinants of vectors of CD in decision-making, management and resource allocation from local (home) to national (country) levels.

In this sense, the present paper uses multi-criteria decision analysis (MDA) to assess vulnerability to vector-borne transmission of *T. cruzi* in domiciliary environments within an area undergoing domiciliary vector interruption of *T. cruzi* in Colombia. The proposed model aims to be viable in terms of cost-benefit. It is applied from the residential level to the national level and is considered sufficiently flexible to be adapted to the reality of CD endemic areas with different epidemiological patterns.

A MDA approach allows situational analysis with a multi-criteria perspective, since a given epidemiological problem is influenced by various characteristics or variables referred to, in this manuscript, as criteria.

The MDA approach is being increasingly implemented in the area of public health (de Oliveira et al. 2015) and has even already been applied to CD (Vinhaes et al. 2014).

## MATERIALS AND METHODS

The following phases of MDA were defined: (i) structuring phase; (ii) modeling phase; and (iii) evaluation phase. The objective of the structuring phase is to understand the studied situation. It comprises four stages: (1) definition of the spatial dimension of the question; (2) generation of multi-criteria tree of indicators and description of the dimensions of indicators; and (3) construction of multi-criteria descriptors. The modeling phase mathematically expresses human vulnerability to vector transmission of *T. cruzi*, and the evaluation phase presents the results of the model.

**Fig. 1:**
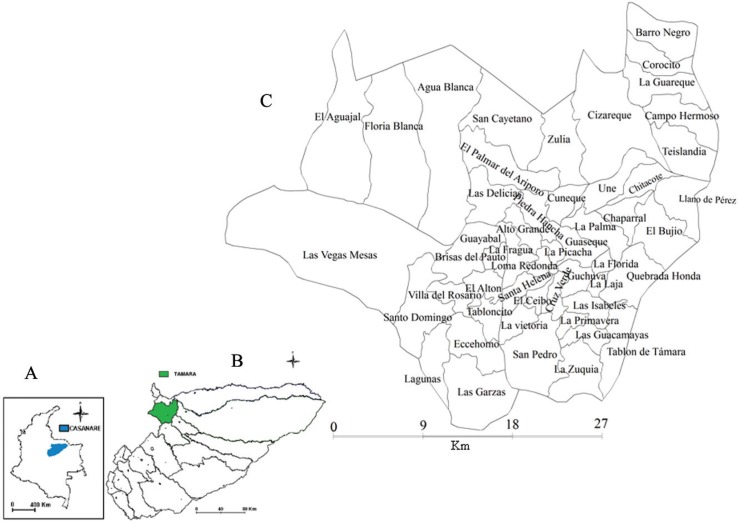
: geographical locations of the department of Casanare (A), the municipality of Támara (B) and the policy division of Támara (C).

*MDA structuring phase* - The spatial dimension in question is defined as the municipality of Támara, located in northwestern Colombia in the department of Casanare (5º49’58.77”N, 72º09’42.05”W). It encompasses a total area of 1,181.81 km^2^, of which 1,180.9 km^2^ are in rural areas containing 50 villages (political divisions) (Fig. 1) (http://www.tamara-casanare.gov.co/informacion_general.shtml). The temperature of the region varies between 12-23ºC (mean 20ºC).

Between 2008-2013, approximately 680 cases of chronic CD were reported in Colombia, plus another 18 acute cases (Vega 2014). Since the first epidemiological reports and studies of CD in Colombia (Guhl et al. 2005), Arauca, Boyacá, Casanare and Santander were recognised as the most epidemiological significant departments (Vega 2014). These departments were subsequently prioritised by the national plan to interrupt the transmission of *T. cruzi* by the intradomiciliar vector *Rhodnius prolixus* (OPS 2011). Within the department of Casanare, the municipality of Támara is considered endemic for CD and was one of three municipalities selected for the implementation of the interruption program (OPS 2011).

A socio-environmental questionnaire and an entomological survey were employed in the municipality (Supplementary data), to evaluate the physical conditions of domicile, the inhabitants’ knowledge about CD, the entomological indicators of triatomines, the presence of hosts/reservoirs of *T. cruzi* and the biotypes of vegetation in the peridomicile area. The techniques and methods used for collecting information were initially presented by Montenegro et al. (2016).

In the multi-criteria tree of indicators and description of the dimensions of indicators, the criteria (variables) were grouped into indicators according to the information reported in Supplementary data, and then included in rates according to the nature of the information. Tree selection follows a logical organisation of criteria and is based on a theoretical framework used by more than 30 previous studies, all of which addressed CD and elements of the transmission cycle of *T. cruzi* (see in detail, Table I). These studies included protocols for triatomine surveillance, meetings and expert consensuses, as well as articles involving field and laboratory work. They also reflect the contribution of more than 100 experts and more than a century of information from CD research, since the time of discovery of CD to the most recent studies of determinants of the disease (Chagas 1909, Montenegro et al. 2016).

**TABLE I:** Evaluation and decision tree for the biological and socio-environmental determinants of vulnerability to intradomiciliary vector-borne *Trypanosoma cruzi* transmission to humans

Indices	Criteria	Estimate	Reference
House indicator (HI)			
Wall type	Bahareque, adobe, wood, block or brick, other, no wall	Percentage composition of each material	(Rabinovich et al. 1979, Guhl 2000, Silveira 2003, Sosa-Jurado et al. 2004, Montenegro et al. 2016)
Wall plaster	Partially, Without plaster, total plaster		
Roof type	Thatch, palm tree leaves, clay tile, zinc, Eternit, etc.		
Floor type	Wooden, tiling, uncoated, cement		
Annexes	Corral: rustic bower, henhouse, stable, pigsty, barn, kitchen, etc.	Qualitative variables: presence and absence	
	Deposits: external areas of leisure or work, accumulation of firewood, stones, etc.		

Triatomine indicator (TI)			
Triatomine reports by the population	Intradomiciliar, peridomiciliar, extradomiciliar	Percentage of homes with triatomine reports	(Silveira et al. 1984, Romaña et al. 1999, MSPS/INS 2011, Abad-Franch 2016, Montenegro et al. 2016)
Presence of triatomines from entomological surveys	Primary species: any domiciled species or population of triatomine (example: *Rhodnius prolixus*)	Entomological triatomine indicators: dispersion, colonization and infestation rates	
	Secondary species: synanthropic species or population that colonize the artificial environment (example: *Panstrongilus geniculatus)*		
	Tertiary species: species or visiting the home environment (example: *R. pictipes)*		
Natural infection with *Trypanosoma* spp.	Primary species	Natural infection percentage with *Trypanosoma* spp by species	(Perlowagora-Szumlewicz & Moreira 1994, Junqueira et al. 2011, MSPS/INS 2011)
	Secondary species		
	Tertiary species		

Ecotope indicator (EI)			
Palm tree	Primary species: palm tree presence functioning as habitat for triatomine specialists or habitat for different species of triatomines. Example: *Attalea butyracea* and *Leopoldina piassaba*.	Percentage of houses with palm tree presence	(Feliciangeli et al. 2007, Noireau et al. 2009, Abad-Franch et al. 2010)
	Secondary species: does not meet the above criteria but can be infested with triatomines. Example: *Elaeis guineesis*, *Mauritia flexuosa*, and other species of *Attalea*)		
	Tertiary species: no history of triatomine infestation. Example: *Euterpe olaracea*		
Presence of other ecotopes (dry trees, bromeliads, nesting birds or mammals)	presence		
Presence of monocultures	presence		

Host/reservoir indicator			
Presence of pets: dogs, @cats, chickens, pigs, etc.	presence	Average number of animals	(Deane et al. 1984, Sosa-Jurado et al. 2004, Noireau et al. 2009)
Presence of *Didelphis* spp	presence		
Presence of other wildlife	presence		

Socio-cultural indicator (S-CI)			
You know it is Chagas disease (CD)	Yes/no	Number of households with knowledge/ relationships/ practices regarding CD	(Tah et al. 2015, Montenegro et al. 2016)
Insect vector transmission associated with CD	Yes/no		
There is practical vector control	Yes/no		
Number of people per house	Value	Average number of permanent residents in the house	

In the construction of multi-criteria descriptors, decision-making processes and indicators of five determinants of the Chagas disease vector pathway were summarily defined, including: (1) house indicator (HI) - an aggregate of different indices regarding the structural conditions of houses; (2) triatominae indicator (TI) - including entomological indices and route of natural infection by *T. cruzi*; (3) host/reservoir indicator (Ho/RoI) - a collection of indices evaluating associations between domestic and wild animals and CD; (4) ecotope indicator (EI) - including several indicators related to surrounding habitats that provide shelter and favor the establishment of triatomine populations; and (5) socio-cultural indicator (S-CI) - a collection of different variables pertaining to population structure and an overview of community knowledge of CD (Table I).

*MDA modeling phase* - In this phase of MDA, indicators, indices and criteria based on numerical values were incorporated into the decision-making model by employing peer-to-peer comparison. This comparison method attributes a preferential value in which two indices, two indicators or two criteria, are graphically compared to one another, generally using the D-sight program (Hayez et al. 2012). All preferential values are detailed in Table I. Theoretical weights, ranging from 1 to 100, for the components of the decision-making model were generated using the preference ranking method for enrichment evaluation (PROMETHHE method) through peer-to-peer comparison (Brans & Mareschal 1994).

It is noteworthy that the PROMETHEE and geometrical analysis for interactive assistance (GAIA) methods were developed in order to help the individual or collective decider. These methods serve to solve problems in selecting or making arrangements of possible alternatives (territories, options, shares), subject to an assessment of various of criteria (variables, qualitative and quantitative indices, indicators, attributes, any criteria with numerical values), which may be in conflict with each other and seek to simultaneously satisfy different views for decision making (Brans & Mareschal 1994, Mareschal & de Smet 2009).

The scores for alternatives (villages) were visualised in frequency histograms for the absolute values of vulnerability, with bars being made to reflect the contribution of each index to the total vulnerability value.

The scores were sorted in ascending order and the villages with the three lowest and three highest vulnerability values were included in the cobweb (radar) graph. The GAIA-stick method was used to visualise vulnerability values of each community previously obtained by the PROMETHEE method (Hayez et al. 2009, Mareschal & de Smet 2009). Visualisation was done at two levels: first, the five indexes were used as statistical vectors, and second, the five indexes were used along with the most relevant vulnerability indicators of the PROMETHEE analysis.

Finally, the resulting scores of the PROMETHEE model were used for spatial statistical analysis. The spatial dependence of vulnerability for each area assessed was analysed using the Moran Local Index - LISA (Anselin 2010), with 9999 permutations, no spatial autocorrelation and considering statistical significance (LISAsig) to be greater than 0.05. All spatial statistical analyses were performed using the program TerraView (INPE 2010).

*MDA evaluation phase* - In this phase, the results of the model are presented.

## RESULTS

Multi-criteria modeling found the determination of vulnerability to vector-borne transmission of *T. cruzi* in the endemic area of Colombia to be mostly influenced by TI with 44.96% of the total weight in the model, while the lowest contribution was from S-CI, with 7.15%. The five indicators are made up of 17 indices, and include 78, of the original 104 priority criteria and variables (Table II).

**TABLE II:** Weights of the criteria for determining vulnerability for vector-borne transmission of *Trypanosoma cruzi* to humans

Criterion	Weight (%)	Absolute weight (%)
Triatomine indicator (TI)	44.96	
Triatomine secondary index	19.88	
Dispertion - *Rhodnius prolixus* (Rp)	7.20	0.64
Colonisation -Rp	52.94	4.73
Peridomiciliary infestion -Rp	15.94	1.42
Intradomiciliary infestion -Rp	23.92	2.14
Triatomine secondary index	19.88	
Dispertion – *Panstrongilus geniculatus* (Pg)	7.20	0.64
Colonisation -Pg	52.94	4.73
Peridomiciliary infestion -Pg	15.94	1.42
Intradomiciliary infestion -Pg	23.92	2.14
Index of perceptions of triatomines	19.30	
Vector perception	7.10	0.62
Palm tree	12.19	1.06
Bush	6.48	0.56
Barn	7.81	0.68
Henhouse	12.06	1.05
Bird nests	8.91	0.77
Stone	8.82	0.77
Firewood	9.02	0.78
Bedroom	19.95	1.73
Kitchen	7.64	0.66
Index infection with *T. cruzi*	40.94	
*Trypanosoma* spp.	100.00	18.41
House indicator(HI)	23.26	
Index of roof	31.50	
Thatch roof	17.61	1.29
Eternit	3.65	0.27
Clay tile	13.39	0.98
Zinc	6.03	0.44
Palm tree leaves	44.30	3.25
Wood roof	15.03	1.10
Index of wall plaster	23.62	
Unplastered	65.48	3.60
Partly plastered	24.99	1.37
Plastered	9.53	0.52
Index of wall	19.57	
Zinc	4.54	0.21
Without wall	22.98	1.05
Wood	11.35	0.52
Block or brick wall	3.88	0.18
Tapia	20.40	0.93
Bahareque	23.87	1.09
Adobe	12.98	0.59
Index of annexes	11.12	
Rustic bower	13.32	0.34
Henhouse	20.60	0.53
Kitchen	13.74	0.36
Stable	18.81	0.49
Pigsty	20.60	0.53
Barn	12.93	0.33
Indicator of floor	14.19	
Wooden floor	22.95	0.76
Tiling	8.72	0.29
Uncoated floor	59.62	1.97
Cement	8.72	0.29
Host/reservoir indicator(Ho/RoI)	12.32	
Wild animals index	16.95	
Primate	14.93	0.31
Rat	19.91	0.42
Mouse	23.04	0.48
Bat	18.89	0.39
Armadillo	23.23	0.48
Index of opossum	41.24	
*Didelphis* spp.	100.00	5.08
Index of domestic animals	41.81	
Equines	16.61	0.86
Pig	28.76	1.48
Chicken	21.85	1.13
Dog	32.78	1.69
Ecotopes indicator (EI)	12.32	
Index of habitats	33.33	
Rock	23.08	0.95
Firewood	23.08	0.95
Forest	20.37	0.84
Bush	17.59	0.72
Trees	15.88	0.65
Cane	16.92	0.35
Coffee tree	26.25	0.54
Banana	15.60	0.32
Crop	15.60	0.32
Grass	25.64	0.53
Index of palm tree	50.00	
Tertiary palm tree	16.34	1.01
Secondary palm tree	29.70	1.83
Primary palm tree	53.96	3.32
Socio-cultural indicator (S-CI)	7.15	
Index of control	37.46	
Unknown	16.61	0.44
Yes	11.52	0.31
Not	71.87	1.92
Index of knowledge of Chagas disease (CD)	33.17	
Other diseases	15.03	0.36
Unknown	8.51	0.20
CD	49.74	1.18
Yes	26.72	0.63
Index of house investigated	29.37	
Average number of persons per household	66.67	1.40
Number of houses	33.33	0.70

According to the weight of each indicator, the largest absolute value for the occurrence of cases of CD by vector are the criteria associated with the colonisation of homes by triatomine vectors *R. prolixus* and *Panstrongilus geniculatus*, natural infection of *T. cruzi* vectors and the perception of triatomines in the bedroom by the inhabitants (Table II).

Houses with palm-leaf roofs or straw walls (bahareque and tapia), without plastering of the walls and with dirt floors were the most critical criteria selected in the HI. The presence of dogs and pigs, and the reports from residents of the presence of *Didelphis* spp., armadillos and mice had the highest contribution within Ho/RoI, while EI was dominated by the presence of rocks, stacked firewood and primarily palm trees in the peridomiciliar area. Distinguishing the pathology as CD and not applying vector control techniques within the S-CI, were the variables with the greatest absolute importance for intradomiciliar transmission of *T. cruzi* (Table II).

**Fig. 2:**
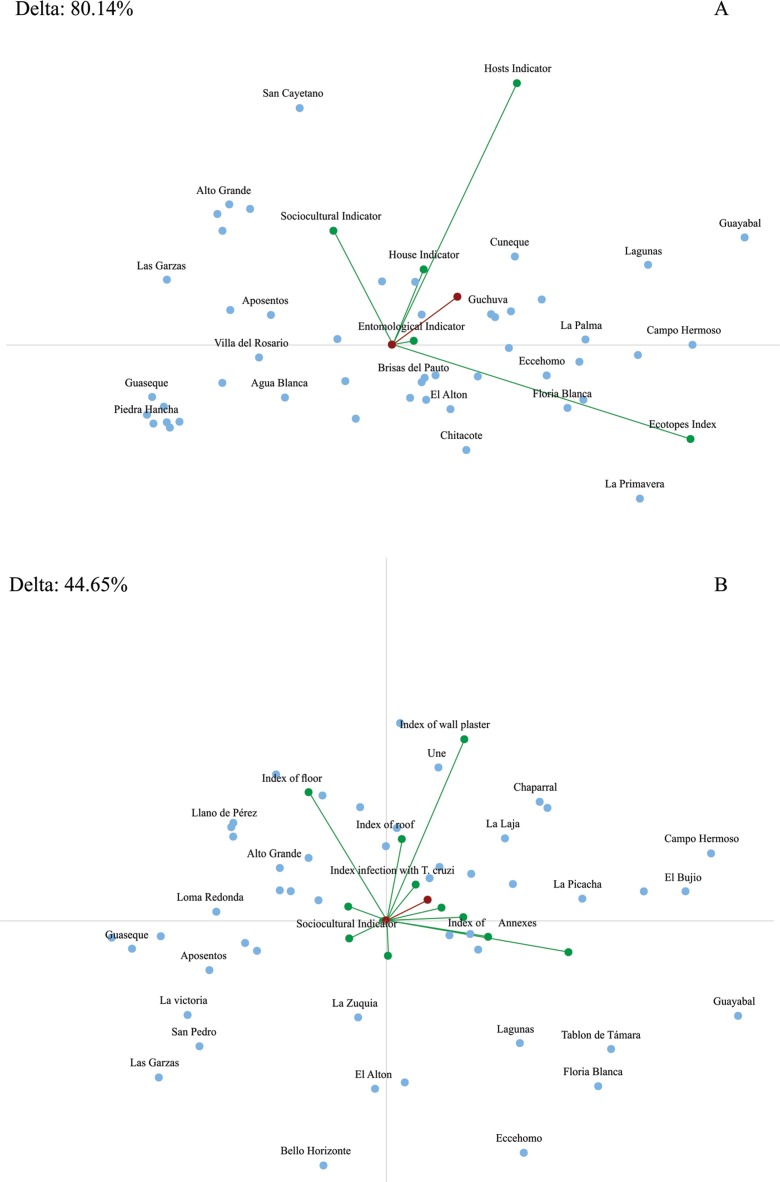
: display of the results of the multi-criteria preference ranking method for enrichment evaluation (PROMETHEE)-geometrical analysis for interactive assistance (GAIA) method for classification of intradomiciliar vulnerability to vector-borne *Trypanosoma cruzi* in an area endemic for Chagas disease. According to the indicator-house indicator (HI), triatominae indicator (TI), host/reservoir indicator (Ho/RoI), ecotope indicator (EI), socio-cultural indicator (S-CI) (B) and indicator and most relevant index of vulnerability (A).

In the multi-criteria evaluation of the results of the PROMETHEE method, heterogeneous vulnerability scores were found for the 46 areas studied, and were normally distributed (standardised skewness and kurtosis of 1.49 and 0.75, respectively). Based on the scores, 23.91% (11/46) of the territories are located in the lowest level of vulnerability (quartile 1), 50.00% (23/46) in the intermediate level (quartile 2), and 26.09% (12/46) in the highest level (quartiles 3 and 4) of vulnerability for the occurrence of CD vector-borne cases (Fig. 2).

**Fig. 3:**
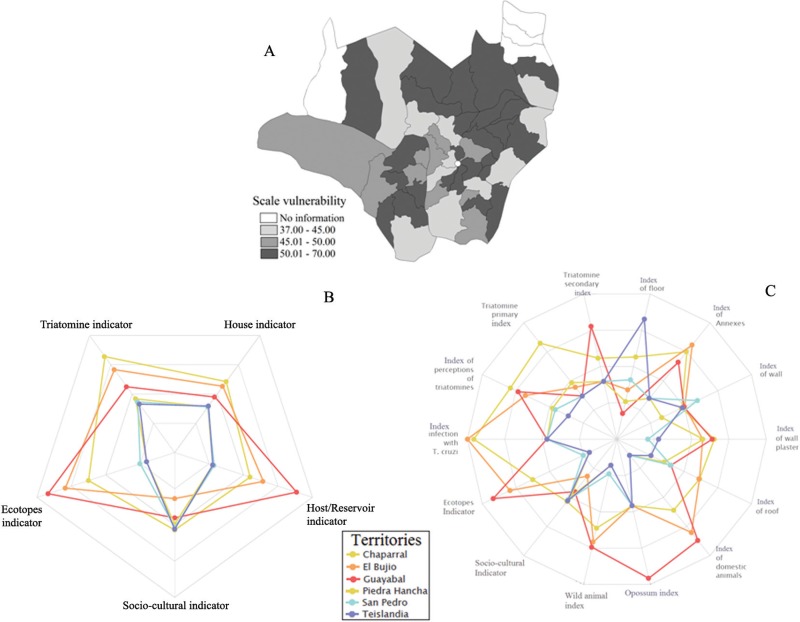
: ranking of the villages according to the preference ranking method for enrichment evaluation (Promethee) for estimating intradomiciliar vulnerability for vector-borne *Trypanosoma cruzi* (A). The three territories with the highest and lowest values of vulnerability based on the indicators house indicator (HI), triatominae indicator (TI), host/reservoir indicator (Ho/RoI), ecotope indicator (EI), socio-cultural indicator (S-CI) (B); the three territories with the highest and lowest values of vulnerability based on the indicators HI, TI, Ho/RoI, EI and S-CI and the most relevant indicators (C).

Territories with the lowest values of vulnerability were Teislandia, San Pedro and Piedra Hancha, while those with the highest were Chaparral, El Bujio and Guayabal (Fig. 3). The most critical are affected by TI, EI and Ho/RoI, and mainly triatomine indices, intradomiciliar perception of insects, natural infection with *T. cruzi,* and the presence of palm trees and domestic animals in peridomiciliar areas. On the other hand, the group with the lowest vulnerability is affected by S-CI, mainly the knowledge and practices of CD vector control (Fig. 3).

The LISA technique identified five villages (La Palma, Quebrada Honda, Santo Domingo, Une and Zulia) with significant spatial autocorrelation, and with it being negative only in Quebrada Honda (-0.0023; p = 0.009) (data not shown).

**Fig. 4:**
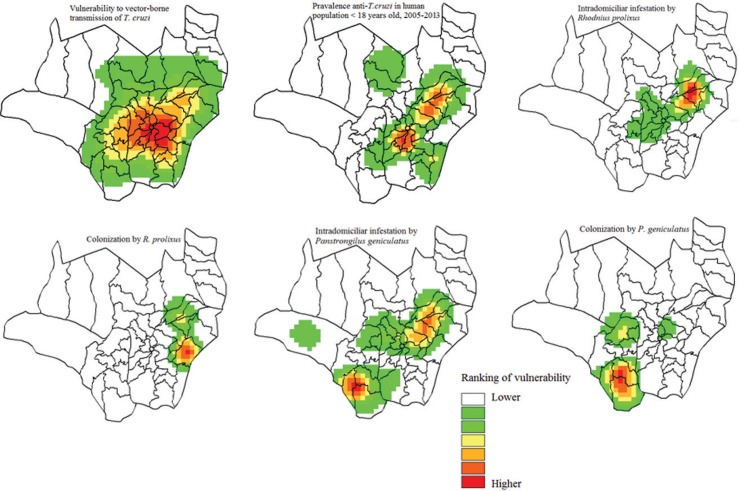
: panorama of human vulnerability to vector-borne *Trypanosoma cruzi* in an endemic area for Chagas disease, 2012-2013.

On the other hand, the GAIA stick method found the TI, with a delta value of 80.07%, followed by the HI, to be the most sensitive statistical vectors for categorising vulnerability of the territories assessed (Fig. 4).

## DISCUSSION

The evidence provided by multi-criteria decision analysis using the PROMETHEE and GAIA methods and spatial statistics can establish scenarios of vulnerability to transmission of *T. cruzi* by different triatomine species in domiciliar environments, and facilitate approaches to combat them.

The PROMETHEE method, characterised as being prescriptive (Mareschal & de Smet 2009), permitted the ranking of villages in order of priority for implementing actions to reduce the vulnerability of human populations to infection with *T. cruzi*. While the GAIA method, characterised as being descriptive and visual, allowed the discovery of the criteria (TI, HI and Ho/RoI) with the greatest contribution to vulnerability to CD via vector through the “stick”-indicator (Fig. 2). The same method also allowed the identification of clusters of communities affected by common criteria (Fig. 2), as has been previously indicated (Hayez et al. 2009). For example, the greater the number of decision criteria used in the analysis, the GAIA method was statistically less robust, from having a Delta value of 80.14% (Fig. 2A), which is considered good (Hayez et al. 2009), to a Delta value of 44.64%. The GAIA method also showed that some criteria, such as S-CI, lose their relevance in stratifying communities by levels of vulnerability (Fig. 2B). This technique works as a principal component analysis (Brans & Mareschal 1994), and so can be useful for prioritising criteria with more or less statistical relevance in decision making.

The technique of spatial statistics, LISA, identified spatial dependence in the establishment of categories of vulnerability in five territories. Spatial analysis methods are useful for the creation or definition of homogeneous areas, the definition of indicators for monitoring and evaluation for a given intervention, and for setting priorities for planning and resource allocation. Therefore, the scenario of neighbor effect (border areas) on local values should also be incorporated in future studies for the establishment of vulnerability for CD.

This work is the first to evaluate the risk assessment questionnaire for Chagas disease in Colombia (Supplementary data) and according to the results, 37% (77/210) of the criteria can be prioritised to categorise houses, villages, municipalities and departments within endemic regions of CD (Table II). However, depending on the eco-epidemiological scenarios in different endemic regions of CD, some other criteria may need to be incorporated for stratification of areas of vulnerability for vector-borne CD.

The model for determining vulnerability presented here is a viable and other alternative to current models of risk (Guhl 2000, Silveira 2003) for several reasons.

This model does not include criteria or variables related to case detection or human infection with *T. cruzi*, which makes it more applicable in regular entomological CD surveillance actions because it can be applied from the residential level to any political-administrative division. Besides, the present work integrated spatial statistics to establish the effect of the neighboring areas in local vulnerability.

The different epidemiological importance of primary (*R. prolixus*), secondary (*P. geniculatus*) and tertiary (*Rhodnius pictipes*) vector species found in the study is related to their degree of synanthropy and the qualitative frequency of incrimination in *T. cruzi* transmission (Table I). This makes the TI flexible and can be adapted to different eco-epidemiological profiles in endemic areas, to the process of interruption and to targeting areas for serological surveillance.

The ideal is to determine which species of triatomine defecate *T. cruzi* metacyclic trypomastigotes, because this is the form infective to mammals (Perlowagora-Szumlewicz & Moreira 1994). However, given the difficulty in differentiating the developmental stages of *T. cruzi*, *Trypanosoma rangeli* and *Blastocrithidia triatomae* within a vector (Junqueira et al. 2011), this study model simply used infection with *Trypanosoma* spp.

Recent classifications of triatomines of epidemiological importance, and relevant monitoring and control plans have been proposed (Abad-Franch 2016).

This study is the first to include the EI, which emphasizes palms. Palms function as an indicator of risk because they serves as habitat for the maintenance of triatomine populations that feed on mammals, birds and reptiles inhabiting the palm trees (Romaña et al. 1999, Noireau et al. 2009, Ricardo-Silva et al. 2012). In this case, we also separate palm trees into primary, secondary and tertiary, depending on the presence in the peridomiciliar area and records of infestation with triatomine. We emphasize the importance of determining rates of infestation with insects of the family Reduviidae (Table I). The presence of these ecotopes (palm trees) in peridomiciliar areas must be prioritised in the processes of interruption, because intradomiciliar infestation by wild populations from palm trees has been shown to occur even after intervention with chemicals (Feliciangeli et al. 2007).

The determinant S-CI, with overall weight of 7.15%, is integrated into the vulnerability model for CD for the first time because it is very important as the pillar for sustaining community participation in the processes of regular entomological surveillance (Tah et al. 2015, Montenegro et al. 2016).

The model of the present study was shown to be predictive in areas where CD cases have historically occurred in the population under 18 years of age, as well as the association between intradomiciliar infestation of *R. prolixus* and *P. geniculatus* and the occurrence of CD cases (Fig. 4). This reinforces that, in addition to *R. prolixus* being the main vector of *T. cruzi* in Colombia, *P. geniculatus* is considered a secondary vector in country (MSPS/INS 2011), and although it was not found to be infected with *Trypanosoma* spp., it may have an important role in the interruption process, as has already been indicated by (Montenegro et al. 2016). This hypothesis is reinforced by the similar values of vulnerability for CD produced by the PROMETHEE method for both species (Table II).

Finally, it is important to mention that the spatial statistical analyses were limited because the data were represented as polygons when data points or homes would have been better. However, this was not possible because at the time of the field survey it was not possible to georeference domiciles due to reasons of public order (presence of armed guerrilla groups).

For some trails it was not possible to work with 100% of the survey responses because some were not completed correctly or lacked relevant information.

Some samples of triatomines provided by community participation were discarded because their location of origin was unknown.

The ideal validation of the present vulnerability model would be by using information from serological screening of a human population for over five years in parallel with environmental and entomological study. Although these activities were carried out jointly, the results of the laboratory analyses were not obtained in a timely manner and/or this information no was accessed.
