# Supplementary material for: Multi-criteria decision analysis and spatial statistic: an approach to determining human vulnerability to vector transmission of Trypanosoma cruzi
Source: Mem Inst Oswaldo Cruz. 2017 Oct;112(10):709–18. doi: 10.1590/0074-02760160523 (PMC5607520; doi:10.1590/0074-02760160523)
Supplement: Supplementary file 1 [file 0074-0276-mioc-112-10-0709-suppl01.pdf]

INSTITUTO
NACIONAL DE
SALUD

Libertad y Orden

DECLARACIÓN DE CONSENTIMIENTO INFORMADO
(Versión preliminar sin revisar, sin validar)

Si usted autoriza su participación y la de su familia en este estudio, por favor complete los siguientes datos.

Yo, ____
Nombre completo de la persona que entrega el consentimiento (jefe de familia o cónyuge)

Declaro que se me han leído y explicado detalladamente los objetivos, los procedimientos y todos los aspectos relacionados con este estudio y que tuve la posibilidad de hacer preguntas para aclarar mis dudas. Acepto voluntariamente mi participación en el estudio y sé que estoy en libertad de retirarme cuando lo desee.

____
Firma de la persona que entrega el consentimiento

Cédula de ciudadanía No. _____________________________________
Verificar con cédula en mano

Fecha ______________________
(dd/mm/aa)

____
Nombre completo del profesional que obtuvo el consentimiento

Firma del profesional que obtuvo el consentimiento: ____

Cédula No: ________________________________

Fecha ______________________
(dd/mm/aa)

Testigo 1

____
Nombre

____
Firma

Testigo 2

____
Nombre

____
Firma

Avenida calle 26 No. 51-20, Bogotá, D.C., Colombia
Conmutador (1) 220 77 00 fax 2200914
Apartados 80080 y 80334
www.ins.gov.co
e-mail ins@ins.gov.co
Línea Gratuita: 018000113400

Nuestra misión: somos un establecimiento público nacional, de referencia científico-técnica, que contribuye a proteger y mejorar las condiciones de salud de las personas, mediante la prestación de servicios y producción de bienes en pro de la salud pública, en el marco del sistema general de seguridad social en salud y del Sistema de Ciencia y Tecnología.

ENCUESTA DE TIPIFICACION DE VIVIENDA
Y FACTORES DE RIESGO PARA LA ENFERMEDAD DE CHAGAS

CÓDIGO ETV ___________________ LOCALIDAD _____________ MUNICÍPIO _____________________ DEPARTAMENTO ________________
ÁREA: Cabecera Municipal ( ) Vereda ( ) Caserío ( ) Inspección de Policía ( )

NOMBRE DEL PREDIO ________________NOMBRE DEL JEFE DE LA CASA _________________ TELEFONO DE CONTACTO: ________________

COORDENADAS DE LA CASA ___________, __________, ____________ FECHA DE RECOLECTA (DD/MM/AA) _______________________
Latitud Longitud Altitud en metros

EPOCA DE MUESTREO: Pre intervención ( ) Post-intervención 24 Horas ( ) Post-intervención 7 Días( )
Post-intervención 14 Días ( ) Fecha última intervención_______________________

ÚLTIMO TIPO INTERVENCIÓN: Insecticidas de acción residual ( ) Pintuinsecticida ( ) Mejoramiento vivienda ( ) Pote Fumigeo ( )
Reordenamiento del domicilio ( ) Control biológico ( ) Control Físico ( ) Toldillos ( )

ULTIMO INSECTICIDAD DE SALUD PÚBLICA APLICADO: Alfa-cipermetrina ( ) Beta-ciflutrina ( ) Ciflutrina ( )
Cipermetrina ( ) Deltametrina ( ) Lambda-cihalotrina ( ) Sin información ( ) Sin aplicación ( )

DATOS FAMILIARES

¿Cuántas personas de su familia duermen permanentemente en la casa por grupo etario?

Sexo/edad	<1	1- 4	5-18	19 - 44	45 - 64	>65	Gestantes
M							
F							

¿Hace cuánto tiempo vive su familia en esta casa? _______ , _______
Años Meses

CONOCIMIENTOS SOBRE LA ENFERMEDAD DE CHAGAS, EL VECTOR Y SU CONTROL

Conoce usted qué es la enfermedad de Chagas? SI ( ) NO ( )

Cómo se llama esta enfermedad?

Enfermedad que es transmitida por un insecto, cuyos síntomas en el humano al comienzo son malestar general: fiebre, intranquilidad, falta de apetito, dolor muscular, vomito, diarrea, agrandamiento de ganglios, hígado y/o bazo hay inflamación de los dos párpados cuando la picadura es cerca del ojo). Cuando la enfermedad progresa produce cansancio al hacer algún esfuerzo, puede aparecer (después de 5 a 20 años) agrandamiento del corazón y puede ser mortal.

Enfermedad de Chagas ( ) No sabe ( ) Otra: ____________

Ha visto este insecto dentro o en los alrededor de su vivienda (mostrar triatóminos)
SI ( ) NO ( )
Cómo se llama estos insectos? a. ( ) triatominos b. ( ) pitos c. ( ) chinches d. ( ) vinchucas e. ( ) barbeiros f. ( ) chipos h. ( ) cuescas i. otros:_____________
. ¿ En qué sitio ha visto estos insectos?
Intra domicilio en: Cocina ( ) Dormitorio ( ) Sala ( ) Comedor ( ) Pasillos ( ) Baño ( )
Otro ( ), Cual____________________
Peri domicilio en: Gallinero ( ) Granero ( ) Monte ( ) Arrume Piedras ( ) Arrume Ladrillos ( ) Arrume de leña ( ) En nidos de aves ( ) Palma ( ) Cactus ( ) otros: __________________________

Extra domicilio en: Cultivos ( ) Bosque ( ) ¿Cuál? ___________________________

Qué enfermedad causa este insecto?
a. ( ) Enfermedad de Chagas
b. ( ) síntomas de picadura (inflamación del parpado (signo de romaña)
c. ( ) no sabe
d. ( ) otro. Cuál? ____________________________________
Métodos de control

La secretaria de salud ha inspeccionado su vivienda?'
d. ( ) Si
e. ( ) No
f. ( ) No sabe
Fecha aproximada de la inspección ______________________
(dd/mm/aa)
La secretaria ha fumigado su vivienda durante los últimos meses?
a. ( ) Si b. ( ) No c. ( ) No sabe Fecha aproximada de fumigación _____________________
(dd/mm/aa)
Ha hecho otro tipo de control en su vivienda durante los últimos meses?
a. ( ) Si
b. ( ) No
c. ( ) No sabe
Cuál fue el tipo de control?
a. ( ) Casero (ordenamiento de la vivienda: limpieza y orden, ventilación, luz)
b. ( ) Reforma de la vivienda
c. ( ) Ambas opciones a y b
d. ( ) Fumigación con insecticida comercial, Cual __________________________________
e. ( ) Otros. Especifique cuál? ____

CARACTERIZACION DE LA VIVIENDA
El tipo de paredes de la vivienda es:
Adobe ( ) Bahareque ( ) Bloque o ladrillo ( ) Madera ( ) Barro pisado o tapia ( )
Sin pared ( ) Otro tipo de pared ( ) ¿Cuál? ________________________

¿Cuál es el tipo de revoque de las paredes?
Empañetada ( ) Parcialmente empañetada ( ) Sin empañetar ( )

¿Cuál es el tipo de techo de la vivienda?
Zinc ( ) Palma ( ) Teja de barro ( ) Teja de Éternit ( ) Techo de paja ( ) Madera ( )
Mixto ( ) Cuál? ___________________ Otro ______________________

¿El techo tiene zarzo (troja)?
SI ( ) NO ( )

El tipo de piso de la vivienda es:
Tierra ( ) Cemento ( ) Baldosa ( ) Madera ( ) Mixto ( ) ¿Cuál? _____________________ Otro, ¿Cuál? _________________________

Anexos de la vivienda
Gallinero ( ) Granero o troja ( ) Pesebrera ( ) Marranera ( ) Conejera ( ) Caney ( ) Horno( ) Trapiche ( ) Otro. ¿Cuál? _____________________

CONTACTO CON POSIBLES RESERVORIOS

¿Escriba el número de cada animal doméstico que duermen en el intra o peridomicilio?
Perros ( ) Gallinas ( ) Marranos ( ) Vacas ( ) Equinos ( ) Gatos ( ) Ninguno ( )
Otro, cuál? _____________________

Ha visto usted alguno de los siguientes animales cerca de la vivienda.
Zarigüeya o chucha ( ) Armadillo ( ) Rata ( ) Ratón ( ) Murciélagos ( ) Conejo ( )
Oso hormiguero ( ) Oso perezoso ( ) primates ( ) Guartinaja ( ) Ñeque ( )
Otro, cual? ______________________

En que sitio los ha visto?
Dentro de la casa ( ) Alrededor de la casa ( ), Distancia (m) _________

En el bosque ( ), Distancia a la casa (m) _________ Otro. ¿Cuál? _______________________

Al observar el alrededor de la vivienda describa el ambiente circundante en un radio aproximado de 100 metros:

Palmas real ( ) Palma Corozo ( ) Palma Africana ( ) Palma Coco ( ) Arbustos ( )
Árboles ( ) Bosque ( ) Pasto ( ) Leña ( ) Piedras ( )

Cultivo, ¿Cuál? _____________________

RECOLECCIÓN DE TRIATOMINOS

Triatominos recolectados (para Ninfas y Adultos ponga el número en la casilla):

Intradomicilio															Peridomicilio															Extradomicilio																			
Dormitorios					Zona social					Cocina					Gallinero					Corral					Troja/deposito					Vegetación					Trampa Noireau			Trampa Angulo			Trampa de Luz		
H	E	N	A	m	H	E	N	A	m	H	E	N	A	m	H	E	N	A	m	H	E	N	A	m	H	E	N	A	m	H	E	N	A	m	N	A	m	N	A	m	N	A	m

H= HUEVOS, N= NINFAS, A= ADULTOS, E= EXUVIAS, m= minutos.

Formulario diligenciado por: ____

Cargo del encuestador: ____
